# Applying the Ugandan Sexual Health and Pastoral Education whole institution approach at the Ugandan UK Health Alliance Summit 2018

**DOI:** 10.4102/phcfm.v10i1.1813

**Published:** 2018-08-08

**Authors:** Emily G. Clark, Sarah Uwimbabazi

**Affiliations:** 1 Royal College of General Practitioners, London, The United Kingdom; 2 Bwindi Community Hospital, Kanungu, Uganda

## Abstract

The Ugandan UK Health alliance (UUKHA) promotes collaboration between UK- and Uganda-based organisations working in health in order to further global learning and the sharing of best practice. On 22–23 March 2018, the UUKHA held the third East African Health Improvement and Investment Summit in Kampala, Uganda. This is the report of a dynamic workshop which was aimed at encouraging thought and discussion on an innovative approach to tackling common health problems such as family planning, alcohol and malnutrition.

## Background

The Ugandan United Kingdom (UK) Health Alliance (UUKHA) promotes collaboration between the UK and the Uganda-based organisations working in health in order to further global learning and the sharing of best practice. On 22–23 March 2018, they held the third East African health improvement and investment summit in Kampala, Uganda. The aim of the summit was to:

- share knowledge on cost-effective innovations to improve health care quality
- strengthen the partnerships between the UK and East Africa in improving the quality; of health care.

Ugandan sexual health and pastoral education (USHAPE) is an initiative run in conjunction with the Royal College of General Practitioners and three hospitals in southwestern Uganda.

The aim of the USHAPE is as follows:

- It disseminates positive messages about modern contraception in an attempt to dispel fears and misconceptions, and identifies and addresses the high rate of unmet need for family planning by screening and training.
- It is different from other family planning (FP) training programmes because it uses a ‘whole institution approach’ so that family planning is no longer just one nurse’s responsibility, and we increase the opportunities patients have to discuss FP and to take up a method. This is designed not only to build the capability and knowledge of staff, but also to provide opportunities and motivation in the working environment to act on that knowledge.

## Aim

The aim of this workshop was to introduce the USHAPE approach to do training, including the concept of the whole institution approach and cascaded outreach to attendees at the UUKHA health summit.

Ugandan sexual health and pastoral education is a successful example of a health partnership that has promoted mutual learning in improving the quality of family planning training and services in southwestern Uganda. It therefore fitted with the main aim of the UUKHA health summit to showcase success in cost-effective health care innovations.

## Methods

Initially, a brief presentation was delivered by Dr Emily G. Clark, a UK general practitioner, and Mrs Sarah Uwimbabazi, a Ugandan nurse, on the whole institution approach and cascaded outreach used in USHAPE family planning training.

In the USHAPE example, the whole institution approach has been applied to family planning. This approach aims to raise the awareness of all clinical and appropriate non-clinical staff in the health facilities about the importance of family planning and opportunities to promote its use. Key staff then have further comprehensive family planning provider training.

This approach of increasing knowledge in a particular subject area for all members of staff at an institution can be applied to other types of public health messages, community health issues and preventative health care topics.

Ugandan sexual health and pastoral education graduates get to put their learning into practice in different types of cascaded outreach. The aim of cascaded outreach is to filter positive family planning messages into the community to increase understanding, dispel myths and thus create a demand for family planning services. It can also provide a point of care counselling and, where appropriate, provision of methods in locations and settings that are relevant to different community groups (men’s groups, church groups, youth groups, etc.). This in turn integrates family planning with other important outreach activities such as human immunodeficiency virus (HIV) counselling and testing, antenatal care, youth-friendly services and male health promotion. It can also provide teaching experience for USHAPE advocates and providers to enhance their knowledge, skills and learning.

Small groupwork around the whole institution approach was then carried out. The group was divided into two smaller groups to imagine a theoretical health care setting. One group was tasked with tackling alcohol use and the other group was tasked with tackling malnutrition.

Both groups were asked to brainstorm:

- How could you use the whole institution approach to look at these issues? For example, who works in your health care setting? In what context would they encounter this issue?
- How do you screen for these issues?
- Who could advocate for these issues?
- How could cascaded outreach tackle this issue?

**FIGURE 1:**
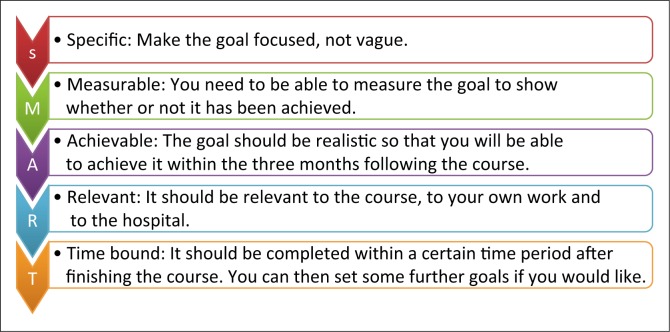
Action planning.

Handouts were provided regarding some of the transferable skills harnessed in the workshop, including action planning (see Figure 1), feedback skills and planning community outreaches.

## Result

There were 17 participants at the 1 h workshop. Participants were from a range of settings across East Africa, including the Ugandan Ministry of Health, Ugandan Midwives Association and Makarere University. The roles of the participants ranged from medical student to quality improvement officer to community nurse.

In the group discussing alcohol, there was discussion on how the different workers at the hospital may encounter alcohol misuse, such as the gatekeeper recognising signs of alcoholism in the boda drivers, and using the opportunity to signpost to direct them to support services for such individuals. There was a discussion about the use of screening tools to screen inpatients and outpatients for alcohol issues.

The group tackling malnutrition discussed the fact that all members of the community and health setting could advocate for this issue by disseminating the positive messages on early treatment of malnutrition through the community and dispelling some of the common myths about the causes of malnutrition that can lead to serious delays in treatment for some children.

At the end, the groups were asked to provide feedback for the session using the feedback tool used in USHAPE training.

They said:

- What went well: The workshop leaders were engaging.
- What could be improved: More time to explore the ideas discussed.
- What went wrong: The alcohol group focussed too much on clinical aspects, so perhaps the exercise was not explained clearly enough to them.
- What would we like to keep: The handouts.
- What did not get enough attention: Putting the action planning tool into practice.

## Discussion

- This was a dynamic workshop which was aimed at encouraging thought and discussion on an innovative approach to tackle common health problems such as family planning, alcohol use, malnutrition and diabetes. The workshop focused on the generic skills of the approach, rather than on the clinical details of family planning.
- In USHAPE, aspects of educational theory and behaviour changing theory have been integrated into all training materials. This workshop incorporated aspects of these skills, such as targeted different learning styles and feedback skills.
- The group brainstorming the topic of alcohol use found it difficult to steer away from focussing on the clinical aspects of alcohol dependence rather than having a more general discussion on the approach. This may represent the traditional didactic teaching styles adopted in health settings across East Africa being more familiar to the participants than the participatory workshop approach.
- Often people will attend a training programme but fail to implement what they have learnt. In order to prevent knowledge and skills from being wasted, the idea of action planning was encouraged at the end of the workshop (see Figure 1). This is a transferable skill that participants could take to other settings.
- Action planning encourages reflection on what people have learnt and provides goals for people to aspire to (see Figure 1). It enables people to break down into specific steps how they will implement what they have learnt. It also enables trainers and mentors to follow their progress towards those goals and hold them accountable. This can be a useful learning and motivational tool towards affecting actual behavioural change following the learning of new knowledge and skills.

